# Synergies and trade-offs in drought resilience within a multi-level UK food supply chain

**DOI:** 10.1007/s10113-023-02046-x

**Published:** 2023-04-04

**Authors:** Dolores Rey Vicario, Ian Holman, Chloe Sutcliffe, Tim Hess

**Affiliations:** 1 Centre for Water, Environment and Development, Cranfield University, Cranfield, Bedford, MK43 0AL UK; 2 Science and Collections Division, Royal Horticultural Society, Wisley, Woking UK

**Keywords:** Risk, Food supply chains, Grower, Retailer, Weather extremes, Irrigation, Reservoir

## Abstract

Weather extremes are the biggest challenge for supply chains worldwide, with food supply chains particularly exposed due to agriculture’s sensitivity to weather conditions. Whilst attention has been paid to farm-level impacts from, and adaptation to, weather extremes, there remains a need to better understand how different actors along the supply chain suffer, react and adapt to these natural hazards and how their resilience-building strategies affect other actors’ and the whole system’s resilience. Taking the UK potato supply chain as a case study, this paper analyses the synergies and trade-offs in drought resilience in a multi-level food supply chain. Data from an online survey (87) and interviews with key informants (27) representing potato supply-chain actors (growers, packers, processors, retailers) were used to analyse drought risk perceptions, impacts and coping strategies, long-term resilience measures and further actions to build system resilience. Results suggest that the potato supply chain has increased its resilience to weather extremes due to retailers and packers having a wider geographical spread of supply, an increasing reliance on forward contracts and favouring growers with water security. However, a conceptual framework of resilience-building strategies adopted by supply chain actors shows that these measures are largely designed to reduce their own risk without considering implications for other parts of the chain and the system as a whole. A more integrated approach to promote drought resilience in complex food supply chains that enables improved vertical collaboration and trust between actors is therefore needed.

## Introduction

Supply chains can suffer disruptions due to a variety of shocks, with adverse weather conditions being the most widespread incident affecting businesses across the world (Allianz Global Corporate & Specialty AG 2012). Extreme weather, including droughts and heatwaves, has caused significant damages globally (Gallic and Vermandel 2020) and affected agricultural production worldwide, leading to financial losses, food supply and food security threats (Gbegbelegbe et al. 2014), increases in global food prices (Brown and Kshirsagar 2015; Malesios et al. 2020) and impacts on producers’ and consumers’ welfare. Given agricultural production’s dependence on weather conditions (Nelson et al. 2014), the projected future increases in the incidence and severity of extreme weather due to climate change (IPCC 2012; EEA 2017; Ault 2020) are likely to increase the challenges for food systems (Gregory et al. 2005; Allouche 2011; Godde et al. 2021). These will be compounded by increasing global population pressures and the increasing scarcity of the natural resources required for producing food (Bates et al. 2008; Hanjra and Qureshi 2010; FAO 2017).

Existing studies on extreme weather and food supply chains mostly focus on present and future impacts on production (e.g. Alidoost et al. 2019), food security (e.g. Gbegbelegbe et al. 2014), prices (e.g. Brown and Kshirsagar 2015; Countryman et al. 2016) and nutrition (e.g. Park et al. 2019). Consideration of actors in the supply chain has largely focused on the ends of the chains, i.e. primary producers (e.g. farms and farmers: Rey et al. 2017) and retailers (e.g. MacFadyen et al. 2015). There is less frequent consideration of intermediary actors within the supply chains such as processors and distributors (de Sá et al. 2019). According to the review on food supply chain resilience to environmental shocks by Davis et al. (2021), around two-thirds of the supply chain coping strategies focus on the production level, disregarding the multiple points where different actors can act to reduce the negative impacts.

Resilience is considered as the ‘capacity to maintain this desired state of food security when exposed to stresses and shocks’ (Ingram 2017) and is conferred by ‘the capacity to anticipate, respond, adapt, or transform’ in response to the stress or shock (Biggs et al. 2021). These capacities may be employed to enhance robustness to the shock (i.e. to maintain the desired state), to recover rapidly after a shock or to reorientate the system to accept alternative outcomes (Zurek et al. 2022).

In their study of the responses of the UK food supply chain to drought, Holman et al. (2021) found that (a) most drought responses were on-farm, although a diverse range of strategies were implemented through the supply chain; and (b) drought responses were dominated by short- and medium-term actions to cope with the drought, with little contribution to future resilience. Pressures from the highly competitive financial environment in which growers operate and uncertainty related to the regulatory and political environment force them to focus on near-term efficiency, preventing many growers from building resilience to water shortages over the longer term (Sutcliffe et al. 2021; Hess et al. 2020; Rey et al. 2017).

The existing literature also tends to focus on individual businesses and organisations within the supply chain rather than considering their position in the chain as a system (Tendall et al. 2015; Hecht et al. 2019; Davis et al. 2021). Due to the large amount of intermediaries in food supply chains, collaboration and coordination is difficult (Yadav et al. 2022). In the UK, Zurek et al (2020) analysed the resilience of fruit and vegetables systems to water related-risks. They found that resilience at an individual actor level does not necessarily result in whole-system resilience. Some of the individual resilience strategies overlap and reinforce each other leading to improved system resilience, but in many cases, there is no coordination between them, as each actor in the supply chain will have their own desired outcomes from the food system, leading to trade-offs and reduced system resilience. For individual growers, resilience may be the ability to produce and sell their crop; for packers and processors, to have consistency in the quality and size of the crop; for retailers, it may be the ability to make a profit from that product. These differences will impact actors’ risk perception and influence their resilience-building decisions (Zurek et al. 2020).

There is therefore a lack of a true supply chain perspective (from production, processing, distribution, marketing through to consumption) in understanding drought resilience and consequently an urgent need to look at the resilience of the whole chain (Macfadyen et al. 2015; Hecht et al. 2019; Meyer 2020) — how actors interact during a shock, how the risks and costs are spread across the supply chain, how they cope and adapt to it. The UKs agri-food sector represents 9.4% of the gross value added (£121.0 billion) (DEFRA 2021), but more than 90% is concentrated downstream of the agricultural sector in manufacturing, retailing and catering, emphasising the need to build resilience to weather extremes in the entire chain and not just primary production.

This paper aims to analyse and evaluate how synergies and trade-offs in individual drought resilience actions affect the system resilience of complex food supply chains, taking the UK potato supply chain as a case study. Through the analysis of the results from an online survey and semi-structured interviews with key stakeholders through the supply chain (from primary production to retail), this paper analyses how supply chain actors cope with droughts and adapt to them in the long term, and how their decisions affect the resilience of other actors and the supply chain. The outcomes, together with a conceptual framework derived from the findings, help to fill the above-mentioned gap in the literature on food supply chains, weather extremes and pathways towards resilience, and will support a systems approach that enables stakeholders across food supply chains to work in cohort to increase the resilience of the system as a whole.

## Materials and methods

### The UK potato supply chain

Potatoes are the most important staple in the UK in terms of production, accounting for ~ 123,000 hectares and annual domestic production of 4–6 million tonnes over the last 3 years (AHDB 2019b; DAERA 2021) with 36.6% going to the pre-pack retail market (AHDB 2019a). The area of potatoes grown for the retail market is the largest, followed by potatoes grown for processing. More than half of the area growing potatoes in Great Britain has irrigation capability (AHDB 2019b), helping to maintain the consistent soil moisture required throughout the growing season to produce high yields and high quality (AHDB 2018). Water abstraction for irrigation requires a licence which can be subject to restriction during dry periods to protect the public water supply and river ecology (Salmoral et al. 2019). Fresh potatoes sales were around £1Bn in 2019, with the value of the potato market (including processed potatoes) being over 50% of the total value of the carbohydrate market (AHDB 2019b). National potato consumption exceeds total production and over a quarter of the UK potato supply is imported (mostly processed potatoes). A small proportion of domestic production is exported (c10%) (Knox and Hess 2018).

**Fig. 1:**
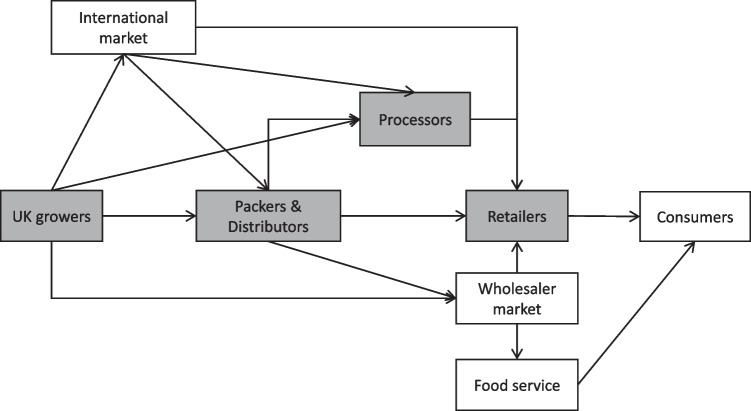
Schematic representation of key actors in the UK potato supply chain. Shaded boxes represent the actors included in this study

The associated enhanced financial capacity needed by growers has seen a transition to bigger and more specialised farms growing potatoes, with a 74% increase in farm size between 2005 and 2019 (AHDB 2019b). The number of registered growers in the UK has also decreased from around 44,400 in 1973 to less than 3000 in 2012, with a 46% reduction between 2005 and 2019 (AHDB 2019b). The supply chain has moved from relying on the open market to having most of the production contracted (81% in 2019) to provide security in potato supply (both in terms of quantity and quality) for packers and retailers for the following season (AHDB 2019a). For growers, such forward contracts provide certainty that they will have a buyer for their product and a known price. However, this contractually obliges them to meet fixed volumes, delivery times and quality specifications, thereby re-enforcing the importance of irrigation and irrigation water security to provide robustness to potential shocks and thereby increasing costs. Crop insurance is not common in the UK (Vyas et al. 2021) with no government-subsidised crop insurance programme (Soil Association 2017). This work focuses on growers, processors, packers and retailers in the UK potato supply chain (excluding wholesaler market, food service and consumers; Fig. 1), and how they are affected by and react to drought risk. Growers are the farmers growing potatoes and selling them to other actors in the chain. Processors are the businesses buying potatoes for processing and selling them mainly to supermarkets (i.e. retailers). Packers buy bulk potatoes and sell them fresh to wholesale, restaurant and food service market or pre-pack to retailers.

UK agriculture has been affected by several drought episodes in the last five decades, affecting crop yields, crop quality, farmers’ income, prices and imports (Holman et al. 2021). The spring of 2011 was one of driest on record in England and Wales, creating challenging conditions for many potato growers who struggled to cope with the water demand of the crop and had to use between a quarter to a third of their total licenced water allocation before the end of May (Knox et al. 2012). Climate change increases the likelihood of these kinds of challenging conditions arising for growers but also represents a potential opportunity for the UK potato industry due to increased potato yields under non-limited water and nutrient conditions, if the food supply chain adapts to meet future irrigation demands (Knox et al. 2011).

### Data collection and analysis

To understand how droughts have affected the UK potato supply chain and how the different actors within the supply chain react and build resilience to this risk, two data collection activities were conducted between November 2017 and April 2018:

- Semi-structured interviews: Semi-structured interviews were carried out with 27 key actors either by phone or face-to-face between November 2017 and April 2018. Although 2018 turned out to be a dry summer (Holman et al. 2021), the drought had not developed by the time of the survey. The 27 participants represented all the actor groups along the supply chain: growers (17, all of them irrigators), processors (2), packers (2), processor-packer (1), packer-retailer (1) and retailers (4). Participants were coded by these terms and a letter (where needed) to anonymise yet retain traceability. Participants were selected using a purposive sampling approach (Robinson 2014) that identified key businesses and personnel in each level of the potato supply chain. Some interviewees were also suggested by participants. The retailers included in the sample represented 59% of the market share in Great Britain at the time of the data collection. We interviewed the major packers and processors that supply fresh and processed potatoes to those big retailers. Potato growers participating in the interviews farmed between 220 and 4500 ha. The combined land area of all participants equalled around 20,000 ha. The semi-structured interviews questionnaire contained open-ended questions to explore the participants’ views on (a) the impacts of low yield and quality on supply chain actors (from farmers to retailers) and coping strategies; (b) the relationship between actors during crisis and (c) long-term resilience strategies, future challenges and ways to increase resilience. The interviews were recorded and transcribed for analysis — transcripts are available via the Data Availability Statement. The data was qualitatively analysed by applying a thematic coding approach (Braun and Clarke 2021) in NVivo 12 (Gibbs 2002), with initial codes related to key themes identified from the literature by the research team, and others emerging from the data collected during the interviews as is conventional within grounded theory literature (Mills et al. 2006). The analysis is both reflexive and iterative ensuring that the qualitative insights were drawn from the data and guided by the literature.
- Online survey: Irrigators in the UK were invited to participate in an online survey on growers’ water risk perception, irrigation practices and commercial arrangements between January and April 2018. From the total sample (*n* = 118), 87 were potato growers and thus included in this study. Survey questions and responses are available via the Data Availability Statement. Descriptive summary statistics were used to summarise relevant results from the survey to complement the qualitative analysis of the interviews.

Both the online survey and the questionnaire were submitted to the University Research Ethics System for approval (CURES/3651/2017 and CURES/1049/2016).

## Results

### Drought risk perception

The growth in irrigation capacity within the potato supply chain has moved perceptions of drought risk away from meteorological and agricultural droughts, which largely impact rainfed potato production, towards concerns regarding hydrological and water resources droughts, which affect the ability of growers to fully irrigate their crops. Water shortages were the top water-related risk for nearly 60% (51) of grower survey participants, with 15 of them reporting drought as being their main concern, and 33 water shortages imposed by the abstraction licencing system. Equally, when discussing water-related risks with growers during the interviews, abstraction licences were also a common concern, both during a drought (when the regulator can mandatorily restrict the water, they can abstract) and in the long term (regarding the reform of the water abstraction licencing system which may lead to reduced licenced volumes or the loss of licences [permits]). For the latter, growers fear losing their spare volumetric capacity (i.e. licence headroom) that helps them cope with dry periods.

During the interviews, a small number of stakeholders mentioned floods as being more severe for their businesses than droughts (5/27) and 3/27 stated it does not matter whether it is a drought or a flood as the consequences are essentially the same — low yields and/or quality issues that will impact actors along the supply chain.

To ensure that we have continuity of supply of the right volume and the right quality of potatoes in a given situation, whether it is a drought or a flood it doesn’t really matter. I suppose potentially a drought is more widespread than a flooding event, but both cause more impact on individual growers. (Processor C)

Over the past 10 years, floods and wet weather are far more of a risk than droughts. We can manage droughts with irrigation, but we don’t have enough drainage capacity to get rid of the excess. (Packer-processor)

Whilst droughts have a direct impact on growers due to the effects on crops, the drought risk perception of other actors down the supply chain diminishes as they are less directly exposed to drought risk. This is because they have a wider range of alternatives coping strategies to deal with drought-related supply shortage (e.g. geographical spread, imports, purchasing product on the open market).

Drought is obviously always on our agenda, but most of our growers have irrigation so…85% of our growers use irrigation and most of them have good licences and they can withdraw from their own reservoir. So, they are fairly well covered. (Packer-retailer).

### Drought impacts and interaction between supply chain actors during a drought

Growers are the first sector in the supply chain to be impacted by the drought, with dry conditions potentially affecting both potato yield (especially for rainfed farms) and quality if there is a lack of water during crucial crop development stages — ‘My assumption would be that farmers are more exposed to the risk. Ultimately if there is a drought in the UK and it affects quality, we are not going to be the only ones affected (or it is unlikely)’ (Retailer A). Increased incidence of common scab (affecting the visual quality of the tuber skin), greening of the potatoes due to exposure to light and high dry matter percentage are typical issues associated to dry weather as reported by respondents. A reduction in yield means growers have less volume to sell and this will inevitably have financial consequences for them. When quality is affected such that retailers will not accept the product, growers can potentially sell them for processing, but at a lower price.

When forward contracts are in place, growers need to forewarn their clients if they cannot deliver the agreed production quantity or meet the quality specifications. Growers always leave a small percentage of their expected production out of the contract as a safety net in case they are short or to sell it to the open market. Despite this, 42 out of the 87 growers participating in the survey admitted that they had been unable to fully deliver on a contract because production was affected by a water-related issue. According to participants, many contracts specify penalties for growers in this situation (22 out of 42 responses, Table 1), but whether or not they are actually applied is variable. The survey results revealed that penalties have been seldom applied as stated by 8 of the non-grower participants, whilst 14 out of the 22 growers who responded to this question reported being affected by penalties. This is somewhat contradicted by the responses from growers participating in the interviews as only 3 of them reported that this happens but it is not normal — ‘Some companies did [impose penalties] and some companies didn’t…’ (Grower M) — and a good relationship and trust seem to be key to avoid those penalties — ‘It is the relationship, the trust between us. I have been in the business for many, many years and I cannot remember having many problems with supply that cannot be worked through’ (Processor B). Ultimately, failing to supply against the contract could mean growers losing their client for the future. Similar conditions and penalties are also applied to other suppliers in the supply chain.

**Table 1:** Survey summary results for questions related to forward contracts (*n* = 87)

Question	Answer	Freq	%
Proportion of production under forward contract	None	3	3.4
	Less than a quarter	5	5.7
	Between a quarter and half	12	13.8
	Between half and three quarters	23	26.4
	Over three quarters	23	26.4
	The entire crop	21	24.1
	TOTAL	87	100
Penalties specified in contract?	Yes	22	25.3
	No	20	23.0
	N/A^a^	45	51.7
	TOTAL	87	100
Penalties enforced?	Yes	14	16.1
	No	8	9.2
	N/A^b^	65	74.7
	TOTAL	87	100

^a^Growers who had never been unable to deliver on a contract due to a water-related problem were not asked this question (includes 3 missing data)

^b^Only growers who answered ‘Yes’ to previous question were asked this question

All actors agree on the importance of having a good relationship with their suppliers and customers to facilitate communication and collaboration in difficult years and minimise contractual difficulties. Many packers/processors/retailers have an agronomist team that is in close contact with their grower base and will identify any problems in the field early in the season. This is crucial for them to find alternative product sources if needed. In this case, they either purchase more products from their current UK growers, if there is any, or go overseas. During past dry episodes, all the non-grower participants relied heavily on imports, mainly from Europe and Mediterranean countries, to compensate for the lack of domestic supply. In addition, a grower representing a big agricultural business reported leaning on the European farms within the business to procure extra production when their UK farm was short — ‘The majority of our supply comes via large, trusted suppliers who work with farms in a broad spread of locations, both UK and internationally. As a result the risk of them being impacted by drought is minimised’ (Retailer A).

When discussing who within the supply chain bears the short-term costs related to drought impacts, different actors in the chain have different opinions. Most of them consider growers as the ones being more affected by drought impacts — ‘The growers stand the costs because they spend more money on irrigation. And the yield will be lower so the cost per tonne will be higher. And in a fully contracted supply chain the grower takes the pain. In a non-fully contracted supply chain then the prices tend to go up and spread evenly across the supply chain’ (Packer B) — The increased costs for growers will to some extent be transmitted along the supply chain, although there were differing opinions between growers (‘I don’t think the consumers carry any of the cost at all, and I don’t think really the retailer carries much cost either. The packer will carry some cost. The grower carries the majority of the cost’ [Grower Q].) and retailers (‘The growers are disadvantaged because they would have less stuff to sell. The suppliers will be disadvantaged because they would have less volume. And the supermarkets are disadvantaged because they would be struggling to meet their customers’ needs and we would have to do other things that will cost us money. It is very unlikely that…nobody is going to be fined, nobody is going to be nasty. It is just they will all take a hit in a different way and usually the end customers will normally have to have a price increase that they don’t like because that will impact their buying so…Everybody takes the hit’ [Retailer B]) regarding the extent to which retailers and consumers are affected.

Drought impacts will ultimately affect consumers, although retailers are very reluctant to increase consumer prices due to very strong price competition between them and the discount supermarkets. The general perception among participants is that consumers do not care how much water is needed to produce the food they buy, and that their choices are mostly price driven. The priority for retailers is to ensure there are potatoes available to customers, and relaxing quality specifications in low supply years has helped keep the shelves full. Consequently, some retailers have invested in promotional campaigns to convince consumers to buy less ‘good looking’ vegetables (e.g. ‘wonky veg’, ‘perfectly imperfect’) in years when visual appearance was affected (NFU 2018).

### Long-term adaptation — building resilience for the future

Interview participants were asked about the main challenges for the sector looking into the future. Whilst climate change is expected to increase the severity and frequency of weather extremes, regulatory uncertainty related to the water abstraction licencing system hinders the ability of growers to plan and invest in long-term adaptation strategies. Abstraction reform was mentioned by both growers and other supply chain actors as an important challenge for the sector. This is because of the increasing importance of both secure access to irrigation water and maintaining licenced headroom to buffer the impacts of a dry year in order to ensure good yields and excellent quality product in the face of increasing climate variability.

Although there were fewer reported examples of longer-term resilience building actions in comparison with coping strategies, and most of them related to on-farm measures, most interview participants think the potato supply chain is more resilient to weather extremes now than in the past. The main adaptation strategies adopted by potato supply chain actors (beyond primary production) as reported during the interviews can be classified around 3 themes: (i) ensuring geographical spread of supply; (ii) increasing reliance on forward contracts and (iii) requiring suppliers’ water security. Processors, packers and retailers are increasing the spatial variety of their grower pools to secure enough supply in the event of adverse conditions — ‘The way businesses review that is not usually every year, but certainly every two or three years. Most big businesses will do a piece of work about where are the potatoes for my supply chain being grown geographically and what is the capability in those areas with those growers. They would do a risk-reward matrix exercise…They do it in different ways, but they all do that type of work. So they have a spread of risk over the geography of the UK’ (Retailer B). This relates to both UK and international supply. Forward contracts are used to reduce the supply risks for all downstream actors as they have an agreed yield and quality for potatoes that will be delivered to them on an agreed date. Finally, retailers, processors and packers are being more selective in their choice of growers, giving preference to those that have reliable supplies of sufficient irrigation water to deal with a drought episode, in particular through investing in on-farm reservoir storage and efficient irrigation technology.

We actually expanded our base because we buy more potatoes from people that we haven’t bought from previously and we keep them on our books and are possibly doing a few more contracts with them. I think we contract a bit more than we used to and we would like to contract a bit more. It gives us a bit more security to make sure that we are more covered than seeing what the yields are each year. So yes, we have tried to mitigate the risk by securing more with contracts but again with people with irrigation and possibly less fluctuation in their yield. So there is a little bit of change there, but not a lot (Packer-retailer).

Despite the perceived improved supply chain resilience, participants suggested further actions to increase the resilience of the supply chain in the future:

i. Improved and increased vertical collaboration between actors within the supply chain — This would help in managing water-related risks. Inspired by a successful initiative with dairy farmers, one retailer has recently created a sustainable farming group for potatoes, whereby they have a direct relationship with a small number of potato growers with the hope to build long-term trusted relationships with them.
  I think we should be developing a partnership with the processors and suppliers and also the growers, to make sure we really understand risk down to specific locations […] Taking a systematic approach to understand what is the risk of drought and the impact that can have on supply. So that is what I think we should do and it is something I am in the process of working through it actually, still very early days (Retailer A).
  From the farmers through to ourselves we realize about the importance of early recognition of a difficult situation, early discussions. And the ability to manage things as a team rather than being aggressive. We pride ourselves and we like to think that we have a good relationship with the growers. They trust us. If we look into the future, I think with pressure on resources, with pressure on agricultural land and more mouths to feed, farming is actually going to be under a lot of pressure, there is going to be a lot of competition there. So you need to develop a very good working relationship with our growers to meet the challenges of the future (Processor B).

ii. Further resilience-building strategies applied by different actors — A range of measures were proposed by participants, but they focused on how growers should adapt to drought risk, rather than looking at other supply chain actors. The most common farm-level adaptation strategy suggested by supply chain actors is increased water storage (excluding growers, 10 in total) and promoting varieties that are more resistant to droughts (3/10); as well as erosion mitigation to prevent water leaving the fields (1/10); and increasing the soil organic matter content to increase its water retention capacity (1/10).
  Taking a systematic approach to understand what is the risk of drought and the impact that can have on supply. So that is what I think we should do […]. I think we are quite blind to the impacts that could have. I think we just rely on our processors and suppliers either having mitigation programs in place, relying on the farmers they use. We don’t have any understanding of how that could impact us (Retailer A).
  Within our grower base we are encouraging farms to be self-sufficient on water, so investment in on-farm reservoirs. Something we need to call for more help from the government in terms of tax break, capital release, easier planning… (Packer B)

However, implementing resilience strategies such as those mentioned above has costs implications — ‘Diversifying supply and having contingency always costs most money and the competitive nature of the UK supply chain at the moment doesn’t encourage you to do that’ (Packer B). In a highly competitive and price-sensitive supply chain, such as the potato supply chain, who should pay for this is a controversial issue that has the potential to hinder future increased investment:

‘The retailers should recognize that food isn’t going to cost less when we have got the extra cost of irrigation. Because we have made a huge investment in reservoirs and in irrigation to produce the consistent quality they require’ (Grower Q).

## Discussion 

### Drought resilience in the UK potato supply chain

Although agriculture is the sector most severely affected by drought, this case study suggests that, despite traditional beliefs, there is burden sharing across the supply chain when it comes to dealing with a shock like a drought event. The risks and the costs associated with this natural hazard affect the supply chain actors in different ways. Supermarkets have increasingly dominated the UK grocery market, with four having a combined market share of over 70% of the sales. Their increased focus on product specification (aesthetic, size and quality standards) can, in many parts of the country, only be achieved with secure and sufficient water availability for supplemental irrigation (Knox and Hess 2018). Over time, this has led many growers to transition from direct summer abstraction of river water for irrigation, to investing in on-farm winter storage reservoirs; from rain guns to more efficient irrigation application systems (e.g. centre pivots, solid state sprinklers, drip irrigation); and to more modern pumping systems and scheduling methods (Rey et al. 2017; Sutcliffe et al. 2021). In this sense, parts of the system have demonstrated a capacity for adaptation in order to enhance the robustness of primary production in response to the risk of drought. This emphasis on robustness over recovery or re-orientation is common in the UK agrifood system (Hess et al. 2020).

Helfgott et al. (2018) encouraged a framing of resilience in terms of resilience ‘of what, to what, from whose perspective and over what time frame’. Both short-term and longer-term drought (‘to what’) management strategies implemented by different actors in the UK potato supply chain are largely designed to reduce their own risk, without considering the implications for other parts of the chain and the system as a whole. Actors were therefore primarily interested in the resilience of the outcomes of their operations (‘of what’) to their business (‘from whose perspective’) and over the short-term (‘over what time frame’). This is consistent with the findings from Peck (2006), when they analysed the resilience of the food and drink supply chains in England and found that business continuity management was applied by organisations driven by self-interest. Thus, there is a high risk of implementing strategies that do not account for the impacts on other actors, which could lead to failure of the desired outcome (Tendall et al. 2015).

### Individual resilience vs. system’s resilience — synergies and trade-offs

Actors respond to shocks within the constraints of the policy environment. The extent to which a company is vertically integrated across multiple steps in a food supply chain also determines options available for them (Davis et al. 2021), with the Internet-of-Things (Ben-Daya et al. 2019) offering the emerging promise of real-time monitoring to support the supply chain in dealing with unpredictable supply variations (Verdouw et al. 2016; Maroli et al. 2021). Existing literature focuses on individual businesses and organisations and how they can build organisational resilience to shocks, rather than looking at how the synergies and trade-offs derived from individual organisation’s resilience strategies affect other actors and the system. Zurek et al. (2020) have shown how the resilience of different parts of the supply chain to drought is intertwined and the ability to absorb a shock in one part of the system can enhance resilience in another.

The framework presented in Table 2 aims at filling this gap for drought risk management in food supply chains, based on the outputs from this study. This conceptual framework identifies resilience-building strategies adopted by actors in the supply chain as well as key external factors that influence resilience, and how they can impact other actors or the whole chain. Synergies are defined as changes introduced by one or more actors in the supply chain that promote their resilience and the resilience of other actors in the supply chain. In contrast, trade-offs are those negative externalities derived from strategies implemented by actors, as well as market and policy factors, that adversely affect the resilience of one or more actors, or even the whole chain. The methods and framework presented in this paper could be adapted to enable a more integrated approach to resilience in other complex food systems.

**Table 2:** Resilience strategies adopted by different actors in the chain, other factors affecting resilience and the synergies (bold) and trade-offs (italics) in drought resilience for different actors and the whole system

	Adopted by				Impact on (synergies and trade-offs)			
**Resilience strategies**	Growers	Packers/ processors	Retailers	External	Growers	Packers/ processors	Retailers	**Whole supply chain**
Forward contracts	More reliance on forward contracts			-	*No benefit from price uplifting*	**Supply and quality assurance**		-
					*Penalties*			
Geographical spread	-	Both in the UK and internationally		-	-	**Access to extra product when supply is low**		*More dependence on water-scarce areas*
								**Supply security**
Water security	- On-farm reservoirs & irrigation technology - Drought resistant cultivars - Soil management	- Preference for growers with irrigation/storage - Use of agronomist teams to identify problems early		-	*Technological lock-in.* *Increased costs*	-	-	-
					**Security in water supply**			
**Other factors affecting resilience**								
High quality standards	-	-	✓	-	*High pressure to use more water* *Increased costs*	-	**Higher prices to be charged to consumers**	*Water resources pressure*
							*Need to find new growers/lose trusted growers*	
Policy changes (abstraction reform)	-	-	-	✓	*Water availability uncertainty*	-	-	*Water resources pressure*
Climate change	-	-	-	✓	*Frequency and severity of shocks*		**Opportunity for new production areas**	*Continuity of supply threatened*
					**Opportunity for growers in currently unsuitable areas**			

One clear example of how resilience-building strategies applied by one sector could put the resilience of the whole chain at stake is the reliance on imports, especially when they come from water-scarce countries (Zhao et al. 2019). In the case of the UK, during low supply years, packers, processors and retailers buy extra potatoes from other countries. During past droughts, potato supply chain actors have been able to switch sources of supply rapidly if required, buying potatoes mainly from Europe. This strategy avoids supermarkets having empty shelves but increases the costs along the supply chain and affects the resilience of the system. The UK relies heavily on food imports from more than 180 countries (DEFRA 2018) which represent around 50% of the food that is consumed (Global Food Security 2019). High import dependency has increased the exposure of the UK food supply chain to water-related risks (Hess and Sutcliffe 2018) and is contributing to deforestation and land use conversion in producer countries (Global Resource Initiative 2020).

Supply chain actors view and respond to extreme events in different ways, which can place conflicting pressures on producers, making adaptation difficult. As derived from Table 2, there are some resilience measures implemented by packers/processors and retailers that could increase pressure on growers and threaten their long-term resilience by: (a) setting high-quality standards that growers need to meet and that require having access to greater volumes of water; (b) forward contracts that prevent farmers from benefiting from price increases during a drought (or other shock that reduces supply) and through which they might be subject to penalties and (c) the need to have on-farm water reservoirs and/or secure access to water to get a contract that could potentially lead to technological lock-in. Also, whilst supermarkets control many aspects of the food supply chain, when it comes to dealing with weather extremes, they necessarily have to rely on growers’ (and processors/packers) resilience.

### Systems-thinking approach to resilience

All the above shows the importance of moving from a silo-thinking approach to resilience to an integrated or connected approach that promotes the resilience of the whole chain rather than only the resilience of the individual actors. With this systems-thinking approach to resilience in mind, there is the need to identify synergetic strategies that would increase the resilience of the whole system and find ways to ensure they are adopted by the relevant actors in the chain. In line with the Protection Motivation Theory (Rogers 1975), there are several factors that will trigger individuals’ decision to protect themselves against a risk like drought. They are categorised as either threat appraisal (i.e. the perceived severity and probability of occurrence of the risk) and coping appraisal (i.e. the perceived self-efficacy to cope with the risk, the response efficacy and the costs). Actors that do not consider droughts as an important risk to their business (threat appraisal) or that do not feel ready to do something about it (coping appraisal) would need different motivation mechanisms to convince them to act upon this risk. Building resilience to weather extremes such as drought carries costs (Holman et al. 2021), which could prevent actors from implementing changes to achieve this, as suggested by the results of this research. For instance, the ability of farmers to finance reservoirs is dependent on the profit margin they can achieve from fixed price forward contracts.

The great complexity of supply chains, plus uncertainties related to climate change and policy suggest that the private sector might struggle to take the appropriate actions (Committee on Climate Change 2019). The multi-level drought management framework of Holman et al. (2021) highlights the importance of non-market institutional arrangements and, in particular, the improved collaboration and engagement across spatial, governance and supply-chain scales that develop human (knowledge) and social (trust) capital in transitioning to longer-term adaptation strategies. There is little understanding of how key actors’ dominant position provides more or less resilience to other actors and to the overall system (Merkle et al. 2021). Political ecology is defined as ‘empirical, research-based explorations to explain linkages in the condition and change of social/environmental systems, with explicit consideration of relations of power’ (Robbins 2012). It explores multi-level connections between global and local phenomena in decision-making and hierarchies of power (Adger et al. 2001). Several authors have already proposed the integration of political ecology and resilience (e.g. Quandt 2016; Beckwith 2022). This approach could help in understanding this conflict between individual and system resilience, as it helps to identify winners and losers, hidden costs, distributional effects and power relationships in social and environmental outcomes (Robbins 2012; Quandt 2016). Governments could play a role in promoting adaptive behaviour through grants, subsidies or tax exemptions for capital investment; or by legislative enablers that promote adaptation and build general resilience (Hess et al. 2020). Public Private Partnerships have been proposed in food systems to solve issues related to public health and food safety (Rouvière and Royer 2017; Fanzo et al. 2020). They could also be implemented to achieving food chain resilience to weather extremes or other disruptions. Adaptation by individual businesses could also be enhanced by other actors in the supply chain, as suggested by Macfadyen et al. (2015) in relation to the fundamental role that retailers can play in promoting the implementation of resilience-prone practices across the food supply chain. Given the concentration of power of the retailers, they might be seen as the critical pivot around which to frame resilience actions (or a key barrier). Increasing the resilience of food supply chains should not be done by producers or policymakers alone (Macfadyen et al. 2015) — all stakeholders, including consumers, have a key role to play.

### Research approach and limitations

This research takes a constructivist approach, where the aim was to understand how different food system stakeholders each construct their own interpretation of the supply chain which then determines their actions in response to drought. The results are based on the thematic coding of their interviews so that the results and discussion reflect the key topics that arose from the interviews and which were then integrated into the framework presented in Table 2. As such, the goal of the research is not to assert an overarching generalisable truth but to understand how and why actors behave differently, and identify the implications of this.

The data collection was done through an online survey and semi-structured interviews with key informants representing different sectors in the UK potato supply chain as described in the ‘Data collection and analysis’ section. Many of these approaches are more usually applied in research of a more positivist/experimental/quantitative nature, rather than our primarily qualitative approach. However, triangulation was achieved by talking to actors at different points in the supply chain and comparing their statements about key topics (e.g. how risk was distributed). Regarding external validity, interviews were undertaken with a large proportion of the ‘population’ of interest — i.e. representatives of all or nearly all the major supermarkets, and the interviewed farmers’ combined landholdings covered a significant portion of the landholdings used for potato production in Eastern and Southern UK.

A more comprehensive data collection, including different actors across the supply chain and ensuring all sectors and products are represented (some actors were not included in our analysis as shown in Fig. 1), could provide a more detailed overview of the synergies and trade-offs to resilience. Also, the combination of this framework with a more quantitative approach could help in assessing the magnitude of those positive or negative impacts on individual and whole system’s resilience.

## Conclusions

According to the World Economic Forum (2019), weather extremes are the current biggest risk for the global economy in terms of impact and livelihood. Weather extremes have caused major disruptions in food supply chains, and this will continue in the future with more severe and frequent episodes. The UK potato supply chain is no exception, having been impacted by drought on several occasions in recent decades. The research has shown that actors along the supply chain, from growers to retailers, have each adopted reactive strategies to limit the impact of the drought on the potato supply to consumers whilst it is happening. These actors are also seeking to implement long-term measures aimed at increasing the resilience of their businesses. However, by analysing the synergies and trade-offs in drought resilience in this case study, this paper highlights how individual resilience strategies can impact other actors in the supply chain and the system’s overall resilience. Most of the measures proposed by participants to further enhance drought resilience of the whole system are focused on growers, but who should pay for the costs associated with these measures remains a contentious issue. Governments and retailers could play a key role in promoting resilience building strategies and supporting their adoption by the different actors in the supply chain. The results highlight that a more integrated approach, involving collaboration and coordination between supply chain actors and between supply and non-supply chain actors, is needed to understand the synergies and trade-offs between individual and systemic resilience building measures in order to promote drought resilience in this and other complex food supply chains.

## Data Availability

The data that support the findings of this study are openly available in Cranfield Online Research Data at: https://doi.org/10.17862/cranfield.rd.14753820.v1 (for survey results); https://doi.org/10.17862/cranfield.rd.12033651 (for growers interviews); https://doi.org/10.17862/cranfield.rd.14761881 (for packers/processors/retailers interviews).
